# *Streptococcus viridans* osteomyelitis and endocarditis following dental treatment: a case report

**DOI:** 10.4076/1757-1626-2-6857

**Published:** 2009-09-14

**Authors:** Maitrayee Choudhury, Brijesh R Patel, Minal Patel, Tariq Bashir

**Affiliations:** 1 CT2 Cardiology, Queens HospitalRom Valley Way, Romford, RM7 0AGUK; 2 FY1 General Medicine, Queens HospitalRom Valley Way, Romford, RM7 0AGUK; 3 FY2 Acute Medicine, Queens HospitalRom Valley Way, Romford, RM7 0AGUK; 4 Department of Cardiology, Queens HospitalRom Valley Way, Romford, RM7 0AGUK

## Abstract

Vertebral osteomyelitis is an uncommon complication of infective endocarditis with the organism *Streptococcus viridans* being a rare cause of the condition. This case highlights an unusual presentation of *Streptococcus viridans* associated with infective endocarditis and pyogenic osteomyelitis in a patient following a dental procedure.

## Introduction

Back pain is a common complaint seen by general physicians. However, causes such as osteomyelitis can occasionally be overlooked. Osteomyelitis is a serious and debilitating condition of which 6% of cases are associated with infective endocarditis, a disease which can have life threatening consequences [1]. Both conditions are thought to develop following spread of a bacteraemic focus via the haematogenous route [2]. This report highlights the case of a patient who presented with back pain and fever following a visit to a dentist to receive minor dental treatment. Blood cultures taken subsequently grew streptococcus viridans and an echo showed mitral valve vegetations. The case thus shows the importance of investigating back pain and looking for associated conditions such as infective endocarditis which can lead to serious consequences if not treated early.

## Case presentation

A 49-year-old Caucasian female shop assistant visited a dental technician to have her teeth cleaned and polished. One week after her visit to the dentist she began to develop severe pain in her lower back. The patient initially saw her general practitioner who initially thought the back pain was mechanical in nature and referred her to an osteopath. However, over the next two months after seeing the dentist the back discomfort worsened and the patient also began to develop paraesthesia over her right thigh. As a result she was admitted to Accident and Emergency department for further assessment of her symptoms.

On admission the patient was haemodynamically stable with low grade pyrexia of 37.5°C. On examination there was tenderness on palpation of the lower lumber spine although there was no overlying erythema or swelling. In addition there were no signs of cord compression and the patient had intact bowel and bladder sensation. Respiratory examination was unremarkable and there were no murmurs noted on cardiac auscultation. The patient had no history of respiratory or cardiac symptoms.

Blood tests revealed a C-reactive protein, (CRP) of 231, erythrocyte sediment ratio (ESR) at 37 mm/h and a white cell count, (WCC) of 7.1 10*9/L. Urea and creatinine were within normal limits. She was noted to be anaemic with haemoglobin (Hb) of 8.3 g/dl and a mean corpuscular volume (MCV) of 82.2 fl. There was no history of menorrhagia or gastrointestinal tract bleeding and she was not on any non-steroidal medication.

**Figure 1.**
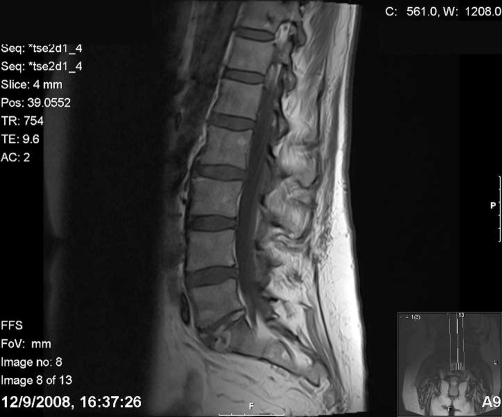
MRI Lumbar Spine showing spondylodiscitis at L5/S1 level.

An MRI was organised within a week following admission to the acute medical assessment unit (Figure 1). It showed increased signal intensity between L5 and S1 vertebrae post gadolin contrast with paraspinous abscess formation, findings which were consistent with spondylodiscitis.

**Figure 2.**
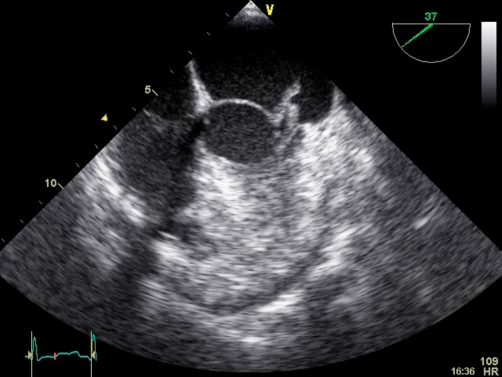
Echocardiogram showing mitral valve vegetation.

Blood cultures taken on admission subsequently grew *Streptococcus viridans* which were isolated from two bottles. The organism was sensitive to Penicillin, Gentamicin, Vancomycin and Rifampicin. In view of the blood culture finding and history of a recent dental procedure prior to the onset of back pain, the patient underwent an echocardiogram. Although no murmurs could be found on auscultation the echo showed a vegetation on the mitral valve with associated mitral regurgitation which was confirmed on transoesophageal echo, (Figure 2). There was no past medical history of valve prosthesis or valvular heart disease.

Following joint discussion with microbiologists, the patient received a 6 week course of intravenous Benzylpenicillin and Vancomycin. Her temperature fluctuated during the course of her stay and the patient did not require any neurosurgical intervention for her spinal osteomyelitis, however, she did receive a 2 unit blood transfusion as her haemoglobin fell to 7.9 g/dl. Following the course of antibiotics, her CRP fell to 13 and haemoglobin improved. She was discharged home with complete resolution of her back pain and outpatient Cardiology and Neurosurgical follow up.

## Discussion

Vertebral osteomyelitis is an uncommon complication of infective endocarditis, however, one retrospective case review looking at cases from 1986 to 2002 has shown a 31% incidence of infective endocarditis in patients with osteomyelitis [3]. The pathogenesis has been said to primarily involve bacteraemic spread via the haematogenous route [4].

Gram-positive organisms such as Staphylococcus aureus and *Staphylococcus epidermidis* have been shown to be the predominant pathogen in pyogenic vertebral osteomyelitis, [3,5]. *Streptococcus viridans* is an unusual organism to be associated with both osteomyelitis and endocarditis with only few studies having reported such a finding [3,5,6]. For example out of 91 patients with osteomyelitis and endocarditis, only 6 out of 25 cases of gram positive organisms grew *Streptococcus viridans*, however, *Staphylococcus aureus* being the most common organism isolated [3]. In our patient the source of infection is likely to have stemmed from haematogenous spread of the organism from dental work the patient received prior to developing back pain. In this case, it is difficult to establish whether the osteomyelitis preceded infective endocarditis a problem which has been noted in previous similar case reports [6,2].

In terms of duration of treatment, the patient received 6 weeks of intravenous (IV) antibiotic therapy as treatment for both the endocarditis and osteomyelitis. One retrospective review found that 25 patients with the condition received a median therapy of 6.5 weeks, ranging from 4 weeks for *Streptococcus viridans* infections and 6 weeks for Staphylococcal infections [5]. Some studies recommend additional oral antibiotic therapy following IV antibiotics based on a review of treatments [1].

Overall, this case further highlights the seriousness of the presentation of sudden onset back pain especially one which is persistent in nature. Our patient had other factors including fever and a raised CRP to indicate an infective aetiology for her symptoms. This is consistent with findings from a study showing that cases of spondylodiscitis secondary to infection by *Streptococcus viridans* tended to have raised blood inflammatory markers but not always have symptoms of systemic upset [5]. However, the insidious nature in which these symptoms can present highlight the fact that life threatening causes can often be overlooked on a general medical unit. In conclusion when assessing a patient presenting with back pain, a thorough history must be taken and osteomyelitis must be considered as a differential as well as associating causes such as infective endocarditis, as these carry a serious risk of mortality if left untreated.
